# Phonological reduplication in sign language: Rules rule

**DOI:** 10.3389/fpsyg.2014.00560

**Published:** 2014-06-10

**Authors:** Iris Berent, Amanda Dupuis, Diane Brentari

**Affiliations:** ^1^Department of Psychology, Northeastern UniversityBoston, MA, USA; ^2^Department of Linguistics, University of ChicagoChicago, IL, USA

**Keywords:** phonology, sign langauge, rules, reduplication, lexical decision

## Abstract

Productivity—the hallmark of linguistic competence—is typically attributed to algebraic rules that support broad generalizations. Past research on spoken language has documented such generalizations in both adults and infants. But whether algebraic rules form part of the linguistic competence of signers remains unknown. To address this question, here we gauge the generalization afforded by American Sign Language (ASL). As a case study, we examine reduplication (X→XX)—a rule that, *inter alia*, generates ASL nouns from verbs. If signers encode this rule, then they should freely extend it to novel syllables, including ones with features that are unattested in ASL. And since reduplicated disyllables are preferred in ASL, such a rule should favor novel reduplicated signs. Novel reduplicated signs should thus be preferred to nonreduplicative controls (in rating), and consequently, such stimuli should also be harder to classify as nonsigns (in the lexical decision task). The results of four experiments support this prediction. These findings suggest that the phonological knowledge of signers includes powerful algebraic rules. The convergence between these conclusions and previous evidence for phonological rules in spoken language suggests that the architecture of the phonological mind is partly amodal.

## Introduction

Productivity is the hallmark of linguistic competence (Chomsky, 1957). English speakers, for instance, routinely extend their linguistic knowledge to novel forms that they have never heard before (e.g., *blogs, emails, sms's*). For generative theories of language, such generalizations immediately suggest that the language faculty encodes abstract algebraic rules (Chomsky and Halle, 1968; Chomsky, 1980; Fodor and Pylyshyn, 1988; Pinker and Prince, 1988; Prince and Smolensky, 1993/2004; Pinker, 1994). But whether rules exist, and whether they are linguistic is a matter of debate.

Dozens of connectionist models have shown that linguistic generalizations can emerge in systems that lack rules altogether (Rumelhart and McClelland, 1986; Elman, 1993; Elman et al., 1996; Seidenberg and Jeffery, 1999; McClelland and Patterson, 2002; Haskell et al., 2003; Bybee and McClelland, 2005; Elman, 2005; Bybee, 2008; McClelland, 2009; McClelland et al., 2010; Ramscar and Dye, 2011). Moreover, all previous attempts to adjudicate between rule- and associative-based accounts have been so far limited to spoken language (e.g., Rumelhart and McClelland, 1986; Fodor and Pylyshyn, 1988; Pinker and Prince, 1988; Marcus, 1998). This lacuna raises the question of whether algebraic rules—if they exist—are specific to spoken communication, or whether they form part of the language faculty, generally.

To address these questions, the present research gauges the role of algebraic rules in American Sign Language (ASL). We begin by considering what algebraic rules are, and how they differ from competing (nonalgebraic) associative mechanisms. We next outline how one can adjudicate between these rival accounts by systematically probing the scope of linguistic generalizations. We first evaluate this question in light of computational and experimental results from spoken languages. These conclusions set the stage for our investigation of rules in sign language.

### Competing accounts of linguistic generalizations: rules vs. associations

To appreciate the anatomy of a rule, let us begin by considering the English plural formation rule as a case study (Pinker, 1999). The plural rule generates plural forms by copying the singular noun stem (N_stem_) and appending the suffix s to its end (N_stem_ + s). This simple description entails several critical assumptions concerning mental architecture (Fodor and Pylyshyn, 1988; Pinker and Prince, 1988; Marcus, 2001; for a glossary, see Box 1). First, it assumes that the mind encodes ***abstract categories*** (e.g., noun stem, N_stem_), and such categories are distinct from their instances (e.g., *dog, letter*). Second, mental categories are potentially ***open-ended***—they include not only familiar instances (e.g., the familiar nouns *dog, cat*) but also novel ones. Third, within such category, all instances—familiar or novel—are equal members of this class. Thus, mental categories form ***equivalence classes***. Fourth, mental processes manipulate such abstract categories—in the present case, it is assumed that the plural rule copies the N_stem_ category. Doing so requires that ***rules operate on algebraic variables***, akin to variables from algebraic numeric operations (e.g., X→X+1)^1^. Finally, because rule description appeals only to this abstract category, the rule will apply equally to any of its members, irrespective of whether any given member is familiar or novel, and regardless of its similarity to existing familiar items^2^. As a result, algebraic rules potentially extend to any member of a class—a property known as ***across-the-board generalizations***.

Box 1 **Glossary.**

- **Productivity**. The capacity to extend linguistic generalizations to novel instances.
- **Across the board generalizations**. Generalizations that extend to any member of a class, actual or potential, regardless of their familiarity or similarity to familiar instances.
- **Algebraic rules**. Mental operations that can potentially extend regularities across the board. These generalizations are supported by various representational capacities, including the capacity (a) to form equivalence classes; (b) to operate on entire classes using variables; and (c) to distinguish types (Noun) from individual tokens (*dog)*.
- **Equivalence class**. A class of elements whose members (actual or potential) are all treated alike with respect to a given generalization. For example, the English plural formation rule (Noun_stem_+s) treats all “noun stems” alike—it applies to either familiar English stems (e.g., *dog*) or novel ones that are nonnative to English (e.g., *ch* in *Chanukah*).

The hypothesis that the language system encodes algebraic rules is consistent with myriad of linguistic data, showing that speakers of many languages extend their knowledge to novel forms. Generalization, however, does not, in and of itself, demonstrate that the mind encodes rules. Indeed, connectionist networks have been shown to exhibit generalizations despite the elimination of algebraic mechanisms—they encode no abstract categories (e.g., Noun) distinct from their instances (e.g., *dog*), and consequently, they lack mechanisms that operate on entire classes (i.e., operations over variables). Generalizations in such models (e.g., to *rogs*) depend not on variables standing for abstract classes (N_stem_ +s), but rather on the association between their specific instances (e.g., between *rog*-*rogs* and *dog-dogs*); the mechanisms that produce regular forms (e.g., *rats*) are indistinguishable from the ones responsible for the formation of exceptions (e.g., *mice*). Yet, such models have been shown to capture significant aspects of speakers' knowledge of existing forms, and even generalize to novel ones (Rumelhart and McClelland, 1986; Elman et al., 1996; McClelland and Plaut, 1999; McClelland and Patterson, 2002).

Given that rules and associations can both lead to generalizations, merely showing that people can generalize linguistic functions cannot adjudicate between competing accounts of language. Nonetheless, algebraic and associationist accounts are not homologous. Their differences become evident once we take a closer look at the scope of generalizations.

### Computational tests of competing architectures: the scope of linguistic generalizations

Algebraic and associationist architectures can both generalize, but the generalizations they attain differ in scope. The evidence comes from computational simulations that systematically gauge the scope of generalizations of a reduplication rule—a function that is commonly found in the morpho-phonology of many languages (e.g., McCarthy, 1986; Suzuki, 1998), and forms the center of our following investigation of sign language. In its simplest form, the reduplication function (X→XX) copies some prosodic unit X (e.g., a syllable; e.g., *baba, dada, tata*). Our question here is what kind of computational system—algebraic or associative—is necessary to freely generalize the reduplicative function to any class member.

Rules, by definition, generalize across the board, so such generalizations are clearly consistent with an algebraic system. A series of simulations by Gary Marcus suggests that they are inconsistent with (nonalgebraic) connectionist networks (Marcus, 1998, 2001). This is not because connectionist networks are categorically unable to generalize; Marcus showed that the reduplication function is successfully learnable by various connectionist networks (feed-forward and simple recurrent networks). But unlike symbolic architectures, generalizations in these networks are systematically limited by the similarity of novel test items to familiar instances.

Novel test items that shared all their features with training instances (i.e., generalizations within their training space) yielded robust generalizations. But when presented with test items including unfamiliar features (i.e., items falling outside the training space), the networks failed to generalize the reduplication function. For example, a network trained on reduplicants with a labial feature (e.g., *papa, mama*) might readily generalize to a novel labial *baba*, as the network can exploit the association between the two labial features in the training items. But since this generalization is solely based on feature-association in training items (e.g., the labial-labial feature), once presented with a velar test item (e.g., *gaga*), generalization will likely fail, as the model lacks knowledge relevant to the reduplication of the velar feature. Subsequent work showed that, absent algebraic rules, the failure to generalize to dissimilar novel items also emerges in the Maximum Entropy Model (Berent et al., 2002)—an influential computational account of phonology (Hayes and Wilson, 2008). Thus, models that lack algebraic mechanisms can generalize, but they cannot do so systematically, across the board.

### The scope of phonological generalizations in spoken language

The systematic links between the architecture of a computational system and its capacity to generalize are significant because they can be used to gauge the architecture of the language system. If the language faculty encodes algebraic rules, then people should extend generalizations across the board, but if they rely on associations, then generalizations will apply only to novel items that share their features with familiar linguistic exemplars.

Previous work on spoken language has tested this prediction using the reduplication function. The evidence comes from speakers of Hebrew—a language that (like other Semitic languages) systematically restricts the location of reduplicated elements in its stems. Hebrew allows identical consonants to occur at the right edge of the stem (e.g., *salal*, “paved”), but bans them in its beginning (e.g., *lalas*; Greenberg, 1950; Leben, 1973; McCarthy, 1986, 1989). Thus, XYY stems (X, Y = any consonant) are well-formed whereas XXY stems are ill-formed.

A large body of experimental research shows that Hebrew speakers generalize this restriction to novel forms (Berent and Shimron, 1997; Berent et al., 2001a,b, 2002, 2004, 2006, 2011, 2012a; Berent and Shimron, 2003)—a conclusion that converges with artificial language experiments with adults (Endress et al., 2005; Toro et al., 2008) and infants (Marcus et al., 1999, 2007; Gervain et al., 2008, 2012). Such results demonstrate that the reduplication function is productive, but they do not attest to the scope of the generalization, and consequently, they do not distinguish between rule-based and associative explanations. Specifically, a generalization to a novel form (e.g., *tagag*) can either occur because Hebrew speakers encode the reduplicative structure of this stem (i.e., as YX_i_X_Ci,_ where the Ci is a copy of element i) or because they associate it with existing stems (e.g., *xagag*, “he celebrated”).

To adjudicate between these competing accounts, one can examine whether Hebrew speakers generalize the identity function to novel stems whose phonemes and features are unattested in Hebrew. For example, Hebrew lacks the phoneme corresponding to the English *th* (e.g., *thing*), and its place of articulation (the wide value of the tongue tip constriction area feature, Gafos, 1999) is likewise unattested. Of interest is whether Hebrew speakers favor novel well-formed YXX stems like *kathath* to their XXY counterparts (e.g., *thathak*, Berent et al., 2002). Findings from a series of experiments suggest that they do just that. Specifically, *thathak*-type forms are less acceptable in rating experiments, and since such ill-formed items are less word-like, they are also classified as nonwords more readily in lexical decision.

The results concerning the reduplication rule are particularly significant because reduplication (and its mirror image, identity restrictions) is fundamental to many phonological and morphological systems (Suzuki, 1998; Frampton, 2009). Accordingly, finding that people extend the reduplication rule across the board suggests that the phonological system of *spoken* language exhibits unbounded productivity—a capacity that would put phonological generalizations on par with syntactic rules. Our present research asks whether algebraic rules also form part of sign language.

### Phonological generalizations in sign language

Every established sign language exhibits a phonological system of intricate design. As in spoken phonology, signed phonological systems encode the hierarchical organization of discrete distinctive features (Brentari, 1998; Sandler and Lillo-Martin, 2006), they represent the syllable—a prosodic unit that is demonstrably distinct from a morpheme (Brentari, 1998; Sandler and Lillo-Martin, 2006), and constrain their sonority profile (Stokoe, 1960; Klima and Bellugi, 1979; Corina, 1990; Perlmutter, 1992; Brentari, 1993, 1994, 1998; Corina and Sandler, 1993; Brentari, 2006; Sandler and Lillo-Martin, 2006; Sandler, 2008; Jantunen and Takkinen, 2010; Wilbur, 2012). Experimental research on sign languages has further shown that signers—both adults (Lane et al., 1976; Newport, 1982; Hildebrandt and Corina, 2002; Emmorey et al., 2003; Baker et al., 2005; Best et al., 2010) and infants (Baker et al., 2006; Palmer et al., 2012)—encode phonological features as phonetic categories, subject to perceptual narrowing in the first year of life (Baker et al., 2006; Palmer et al., 2012). Moreover, distinct feature classes differ in their contribution to language processing. Location information, specifically, is particularly salient to lexical access (Emmorey and Corina, 1990; Corina and Hildebrandt, 2002; Thompson et al., 2005; Baus et al., 2008; Carreiras et al., 2008; Orfanidou et al., 2009; Gutiérrez et al., 2012); it provides a strong cue for similarity (Hildebrandt and Corina, 2002; Bochner et al., 2011); and it is acquired earlier (Siedlecki and Bonvillian, 1993) and more accurately (Marentette and Mayberry, 2000; Morgan, 2006) during first-language acquisition. Other studies have suggested that typical (Morgan, 2006; Morgan et al., 2007) and disordered (Marshall et al., 2006) acquisition of sign language is constrained by the complexity of features and their distance from the body (Meier, 2000; Meier et al., 2008)—a factor also affecting adult signers (Poizner et al., 1981).

Most of this work, however, has focused on individual phonological features, rather than the restrictions governing their combination, and with a couple of exceptions (Carreiras et al., 2008), most results obtained from existing signs. There is also some evidence that signers are sensitive to phonotactic legality (Orfanidou et al., 2010) and the number of syllables in novel signs (Brentari et al., 2011)—phonological units distinct from morphemes (Berent et al., 2013). Nonetheless, it is uncertain whether such knowledge reflects algebraic rules, or the statistical structure of the lexicon—a factor to which signers are acutely sensitive (Carreiras et al., 2008). Whether signers possess the capacity for unbounded productivity—the hallmark of powerful algebraic mechanisms—is unknown. No previous experimental research has addressed this question.

Only one previous study examined the capacity of 7.5 month-old hearing infants to acquire rules from novel signs (Rabagliati et al., 2012). The results, however, were mixed. While participants in this experiment freely extended the YXX rule, they failed to acquire the XXY regularity—a rule they can readily learn from speech stimuli. Moreover, the (limited) generative mechanisms available to infants might not necessarily form part of the linguistic competence of adult signers. One thus wonders whether algebraic rules are inherent to the phonological mind (Berent, 2013a), generally, or to the speech modality, specifically^3^.

### Our present experiments: do signers extend the reduplication function across the board?

Our present study examines the scope of phonological generalizations of the reduplication function. We chose the case of reduplication for two reasons. First, reduplication has been the subject of intense computational effort, so the principled limitations of nonalgebraic mechanisms to extend this function are well documented. Second, reduplication is central to the phonology and morphology of sign language. Like spoken phonological systems, signed phonological systems exhibit various forms of reduplication (Klima and Bellugi, 1979; Sandler and Lillo-Martin, 2006; Wilbur, 2009). One such form generates ASL nouns by reduplicating their verbal counterparts—this process maintains the handshape, location and directionality of movement of their base verb, but invariably changes the frequency and manner of movement to become restrained and repeated (Supalla and Newport, 1978)^4^. While this relationship is systematic (Wilbur, 2009), the class of such verb-noun pairs is rather small, and it is unknown whether it is productive (i.e., whether it generalizes to novel signs). Indeed, related research on reduplication in sign language acquisition (Morgan, 2006) has invoked motor, rather than cognitive factors (Meier et al., 2008). Our following research thus asks whether signers (and nonsigners) extend this rule productively, and whether they do so across the board—regardless of whether the reduplicated feature is attested in their language.

Experiments 1–2 present participants with novel disyllabic signs—either reduplicated or nonreduplicated controls, matched for the first syllable. Using X and Y to represent those two syllables, reduplicated and nonreduplicated signs can be denoted as XX and XY, respectively. These syllables are comprised of native ASL features, and their phonotactic structure is otherwise legal. If signers encode the reduplication rule, then they should favor novel reduplicated signs to their nonreduplicated counterparts. Such preference is expected either because reduplication is grammatically better-formed (i.e., unmarked^5^; McCarthy and Prince, 1995) or because, as a type, reduplicated signs are far more frequent in ASL than nonreduplicated disyllables. Either way, XX novel signs should appear more “sign-like.” Accordingly, novel XX signs should be rated higher than XY controls, and they should be harder to classify as “nonwords” in lexical decision. Experiments 1 (rating) and 2 (lexical decision) address these questions.

The hallmark of algebraic rules, however, is that they support generalizations to *any* member of a class—actual or potential, and past research documented such generalizations in spoken languages. Experiments 3–4 next ask whether unbounded productivity also applies to signs. Experiment 3 elicits ratings of reduplicated signs with unattested handshapes; in Experiment 4, participants perform lexical decision. If reduplication is represented by an algebraic rule, then XX forms should appear more sign-like even when the reduplicated form includes an unattested feature.

## Part 1: generalization to attested features

### Experiment 1: off-line rating

**Figure 1:**
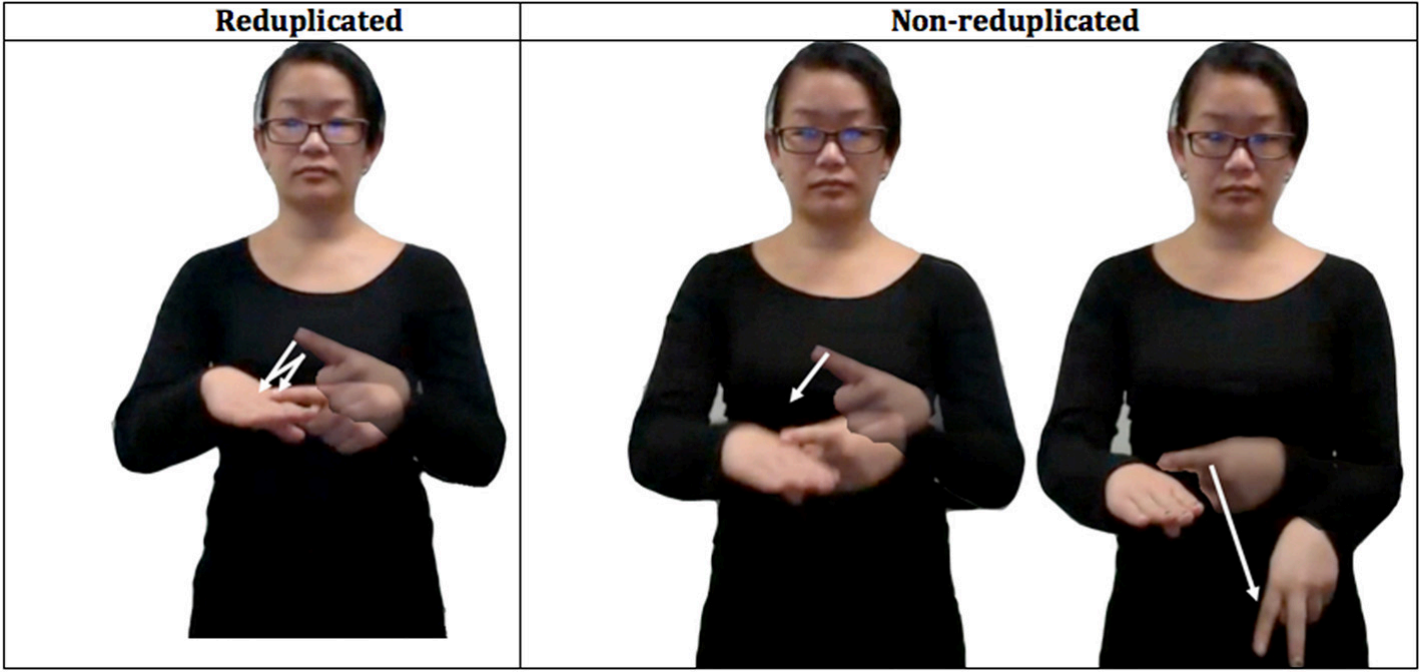
**An illustration of the novel signs used in Experiment 1**.

As a preliminary test, Experiment 1 evaluates signers' sensitivity to reduplication using an off-line rating task. In each trial, participants are presented with a pair of video clips featuring novel ASL signs—a reduplicated XX sign and a nonreduplicated XY control—matched to the reduplicated sign for the initial syllable X (see Figure 1). Of interest is whether signers favor novel reduplicated signs to XY controls.

To determine whether this preference is modulated by linguistic experience with ASL, we also elicited similar ratings from a group of nonsigners, native English speakers. Convergence between the two groups will suggest that the effect of reduplication solely stems from sources (linguistic or otherwise) that are independent of linguistic experience with ASL; divergence will suggest that the encoding of reduplication is at least partly modulated by linguistic knowledge.

#### Methods

***Participants.*** Two groups of adult participants took part in the experiment. One group consisted of twelve Deaf signers who were all exposed to ASL by the age of five (three were exposed to ASL from birth, four by the age of two, and the remaining five by the age of five). The second group consisted of twelve English speakers who were not signers of ASL. Eleven of these participants reported no previous exposure to ASL; one participant had a rudimentary knowledge of the ASL alphabet.

***Materials.*** The materials consisted of short video clips, featuring sixteen pairs of novel disyllabic signs. Within each pair, one member was reduplicated (XX), whereas the other member was nonreduplicated (XY). Pair members were matched for the first syllable (X) and they were phonotactically legal in ASL. A complete list of the materials is presented in Supplementary Material.

These materials were video recordings of a native ASL signer. Prior to the recording, the signer practiced the items so that they are signed naturally. Another native ASL signer recorded the instructions to the experiment in ASL. The video recordings of the stimuli were subsequently edited, so that each video clip began immediately upon the initiation of the signing movement and ended with the signer returning to a neutral position. All video clips were inspected for clarity by a fluent ASL signer (DB).

***Procedure.*** In each trial, participants were presented with a matched pair of novel signs (XX and XY, counterbalanced for order). Signers were told that while the stimuli are not ASL signs, they could potentially exist in ASL. Nonsigners received the same instructions, with the added acknowledgement that the task is difficult to perform without knowledge of ASL and the request to “just try to go with your gut feeling.” Participants were asked to indicate which pair member is more acceptable as an ASL sign. They were allowed to replay the two options as necessary. Signers were presented with the instructions in ASL, whereas nonsigners were presented with English instructions. In this and all experiments, trial order is randomized.

#### Results and discussion

**Figure 2:**
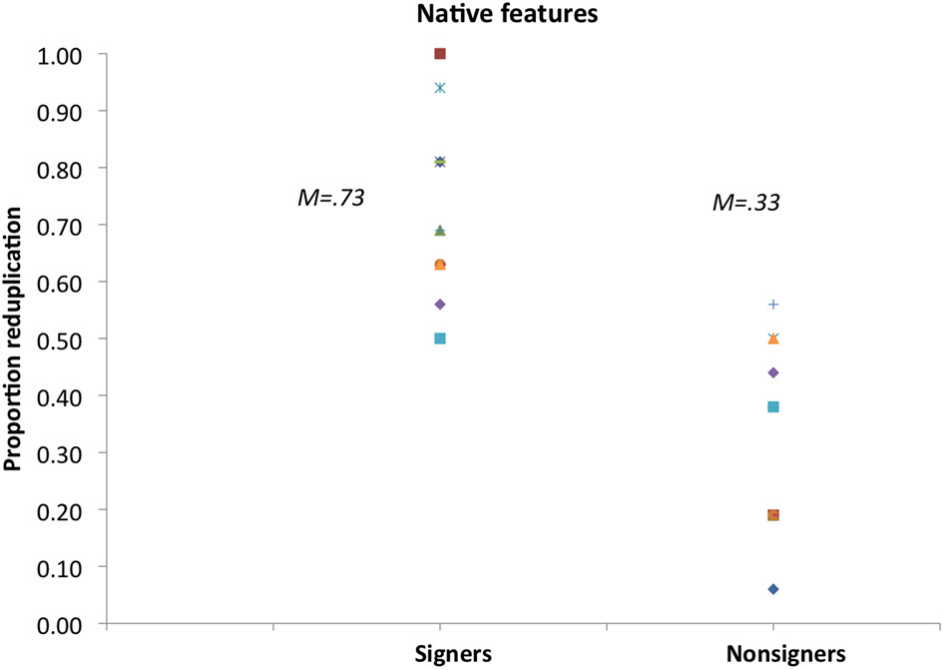
**Rating preference for reduplicated signs with native ASL features in Experiment 1**.

Figure 2 plots signers' rating preferences. Results show that on most trials (73%), signers favored reduplicated novel signs to nonreduplicated controls, and these ratings were found statistically different from chance by *t*-tests [*t*1_(11)_ = 5.16, *p* < 0.003; *t*2_(15)_ = 6.04, *p* < 0.0001]. Nonsigners, by contrast, exhibited no such preference. In fact, nonsigners favored nonreduplicated to reduplicated signs [*M* = 33%, *t*1_(11)_ = −3.68, *p* < 0.006; *t*2_(15)_ = −5.44, *p* < 0.0001].

Signers' capacity to extract reduplication from novel signs is consistent with the possibility that they rely on an algebraic rule. The contrast between the performance of signers and nonsigners suggests that this rule is informed by their linguistic experience with ASL.

The results from the off-line rating procedure, however, are limited inasmuch as they do not address the role of rules in on-line language processing. To examine this question, we next turn to investigate whether signers might encode the reduplicative structure of signs when a rapid on-line response is required, using the lexical decision task.

### Experiment 2: lexical decision

Experiment 2 probes signers' sensitivity to reduplication in the lexical decision task. In each trial, participants were presented with a video clip featuring either an attested ASL sign or a novel sign. Within each such category, half of the items exhibited reduplication (XX), whereas the other half was not reduplicated (XY). Participants were asked to quickly determine whether the stimulus is a real ASL sign, and indicate their response by pressing one of two keys (1 = ASL signs; 2 = nonsigns).

If signers can extend the reduplication rule productively, then reduplicated XX signs should be differentiated from nonreduplicated XY controls; and since novel XX signs are grammatically structured and better formed (i.e., unmarked as compared to XY forms), then they should further appear as more sign-like. Consequently, novel XX signs should be harder to classify as nonsigns relative to nonreduplicated XY controls. In contrast, attested ASL signs with reduplication should be classified more readily than their XY counterparts.

#### Methods

***Participants.*** Participants were the same individuals who took part in the rating experiment (Experiment 1), administered after Experiment 2. Data from one of these participants were excluded from all analyses of Experiment 2 because this individual had reported that he did not understand the task after completing the experiment—an assessment consistent with this individual's accuracy (45%). The results are based on the data of the remaining eleven participants.

***Materials.*** The materials consisted of 16 pairs of ASL signs and 16 pairs of novel ASL signs. Within each category, half of the items were reduplicated, whereas the other half was not reduplicated. The reduplicated ASL signs were all disyllabic nouns that are morphologically related to an ASL verb^6^. The nonreduplicated ASL signs were all ASL compound signs. The reduplicated and nonreduplicated pair members were matched for either handshape (in 6/16 pairs) or location (in 10/16 pairs). Novel signs corresponded to the same novel signs used in Experiment 1. All signs (attested ASL and novel) were recorded by the same native signer. The video recordings of the stimuli were subsequently edited, so that each video clip began immediately upon the initiation of the signing movement and ended with the signer returning to a neutral position. All recordings were inspected for clarity by a fluent ASL signer (DB). The complete lists of the novel and existing ASL signs are presented in Supplementary Material.

***Procedure.*** Each trial began with a screen displaying a fixation point. Participants initiated the trial by pressing the spacebar, and their response triggered the presentation of a single video clip (for up to 4 s). Participants were informed that they were about to watch videos of real and novel signs in American Sign Language. They were told that the novel signs are not used in ASL, but they potentially could be “true ASL signs.” Participants were asked to determine whether the stimulus was a real ASL sign, and indicate their response by pressing one of two keys (1 = sign, 2 = nonsign). They were instructed to make their response as quickly and as accurately as possible. Slow responses (slower than 2250 ms) triggered the presentation of a warning message (an image of a clock), reminding participants to respond faster. Likewise, participants received computerized feedback on their accuracy (green “smiley” face vs. red “sad” face for correct vs. incorrect responses, respectively).

Prior to the experiment, participants took part in a brief practice session. None of the practice items appeared in the experimental session. In this and all subsequent experiments, response time is reported from the onset of the stimulus.

#### Results

**Figure 3:**
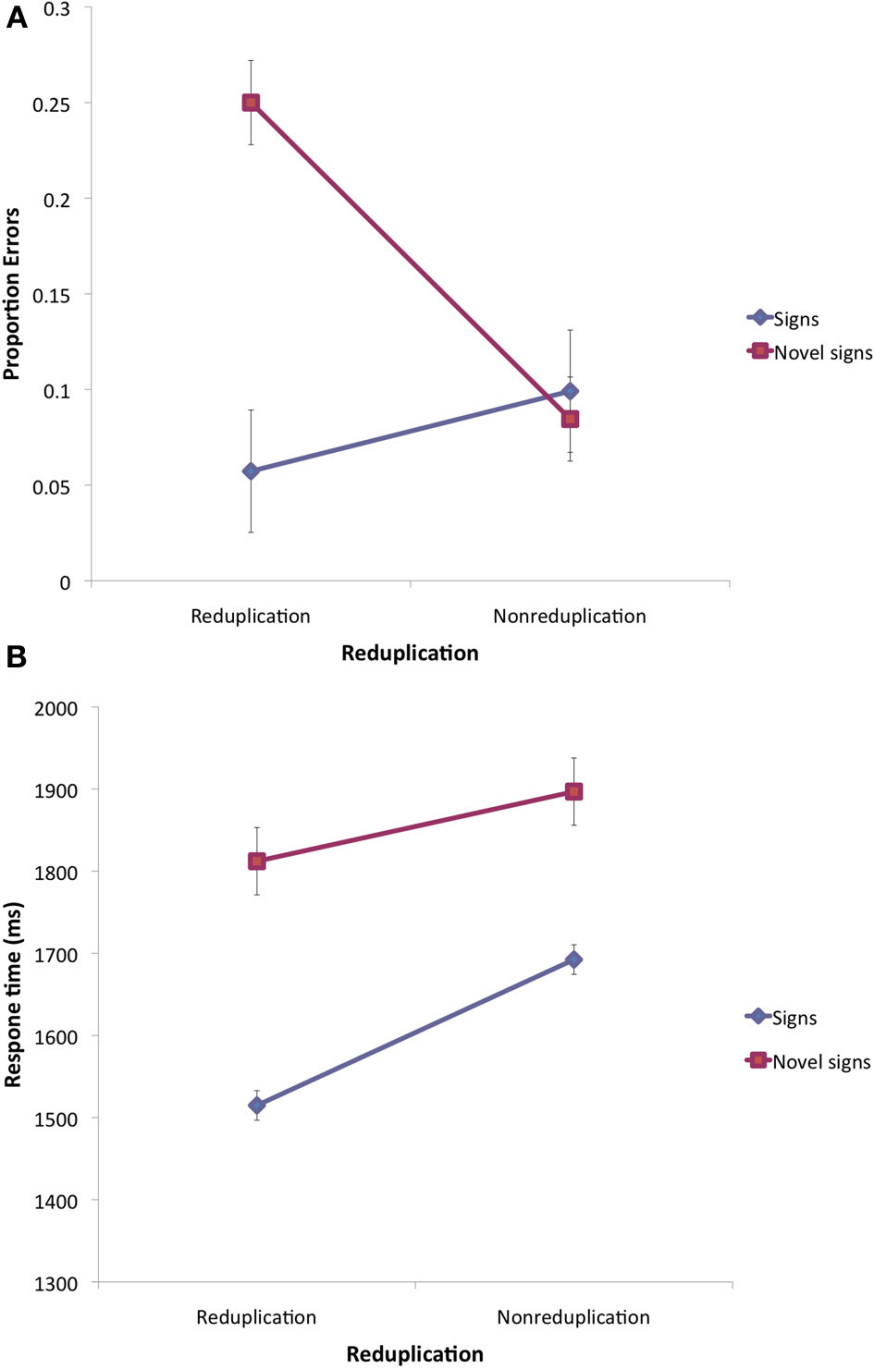
**Lexical decision results for ASL signs and novel signs with native ASL features in Experiment 2**. Note: Error bars are 95% confidence intervals for the difference between the means.

Outliers (correct responses slower than 3000 ms or faster than 250 ms, less than 1.6% of the total correct responses) were excluded from the analyses of response time. The mean error and correct response time of signers to ASL signs and novel signs is presented in Figure 3.

***Errors.*** An inspection of the error means suggests that signers were sensitive to reduplication. Reduplication elevated errors in response to novel signs, but tended to improve accuracy for existing ASL signs.

These conclusions were supported by the 2 lexicality (sign vs. novel sign) × 2 reduplication (reduplication vs. nonreduplication) ANOVAs, conducted over the error data (arcsine transformed) using both participants (F1) and items (F2) as random variables. The analyses yielded a significant main effect of lexicality [*F*1_(1, 10)_ = 8.10, MSE = 0.058, *p* < 0.02; *F*2_(1, 30)_ = 5.48, MSE = 0.167, *p* < 0.03] and a marginally significant effect of reduplication [*F*1_(1, 10)_ = 2.97, MSE = 0.027, *p* < 0.12; *F*2_(1, 30)_ = 3.11, MSE = 0.07, *p* < 0.09]. Crucially, the interaction was highly significant [*F*1_(1, 10)_ = 15.63, MSE = 0.065, *p* < 0.003; *F*2_(1, 30)_ = 9.28, MSE = 0.075, *p* < 0.005]^7^.

To further probe this interaction, we next tested the effect of reduplication for ASL signs and novel signs, separately. Novel reduplicated signs produced significantly more errors compared to nonreduplicated controls [*t*1_(10)_ = 5.68, *p* < 0.0003; *t*2_(15)_ = 4.78, *p* < 0.0003]. The opposite trend emerged for signs, but it was not significant [*t*1_(10)_ = 2.00, *p* < 0.08; *t*2_(15)_ < 1].

***Response time.*** Figure 3 provides the mean correct response time as a function of lexicality and reduplication. The 2 lexicality × 2 reduplication ANOVAs yielded only a reliable main effect of lexicality [*F*1_(1, 10)_ = 101.22, MSE = 6839, *p* < 0.00001; *F*2_(1, 29)_ = 45.16, MSE = 19,450, *p* < 0.0001] and reduplication [*F*1_(1, 10)_ = 29.07, MSE = 6510, *p* < 0.0004; *F*2_(1, 29)_ = 13.63, MSE = 2216, *p* < 0.002]. The reduplication × lexicality interaction was marginally significant [*F*1_(1, 10)_ = 4.77, MSE = 4949, *p* < 0.06; *F*2_(1, 29)_ < 1].

Tests of the simple main effect showed that reduplicated signs elicited reliably faster responses compared to nonreduplicated signs [*t*1_(10)_ = 9.54, *p* < 0.0001; *t*2_(15)_ = 3.64, *p* < 0.004]. In contrast, for novel signs, the effect of reduplication was not reliable [*t*1_(10)_ = 2.04, *p* < 0.07; *t*2_(14)_ = 1.76, *p* < 0.11].

#### Discussion

Experiment 2 examined whether ASL signers extend the reduplication rule to novel signs. Because reduplicated stimuli are grammatically structured, we expected novel reduplicated signs to appear more sign-like. In accord with this prediction, novel reduplicated signs produced more errors, suggesting that they resemble ASL signs more than nonreduplicated controls. In contrast, for existing ASL signs, reduplication sped up response relative to nonreduplicated controls. These findings demonstrate that participants are sensitive to the reduplicative structure of novel signs—an observation consistent with the hypothesis that signers encode productive grammatical rules.

## Part 2: generalization to unattested features

Experiments 1–2 suggest that signers can extract the reduplication of signs whose features are all native to ASL. The hallmark of algebraic rules, however, is that they support broad generalizations to *any* class member. Accordingly, if signers encode reduplication by a rule (X→XX, where X stands for any syllable), then they should extend it not only to novel syllables with native ASL features (studied in Experiments 1–2) but even to novel syllables with unattested phonological features.

**Figure 4:**
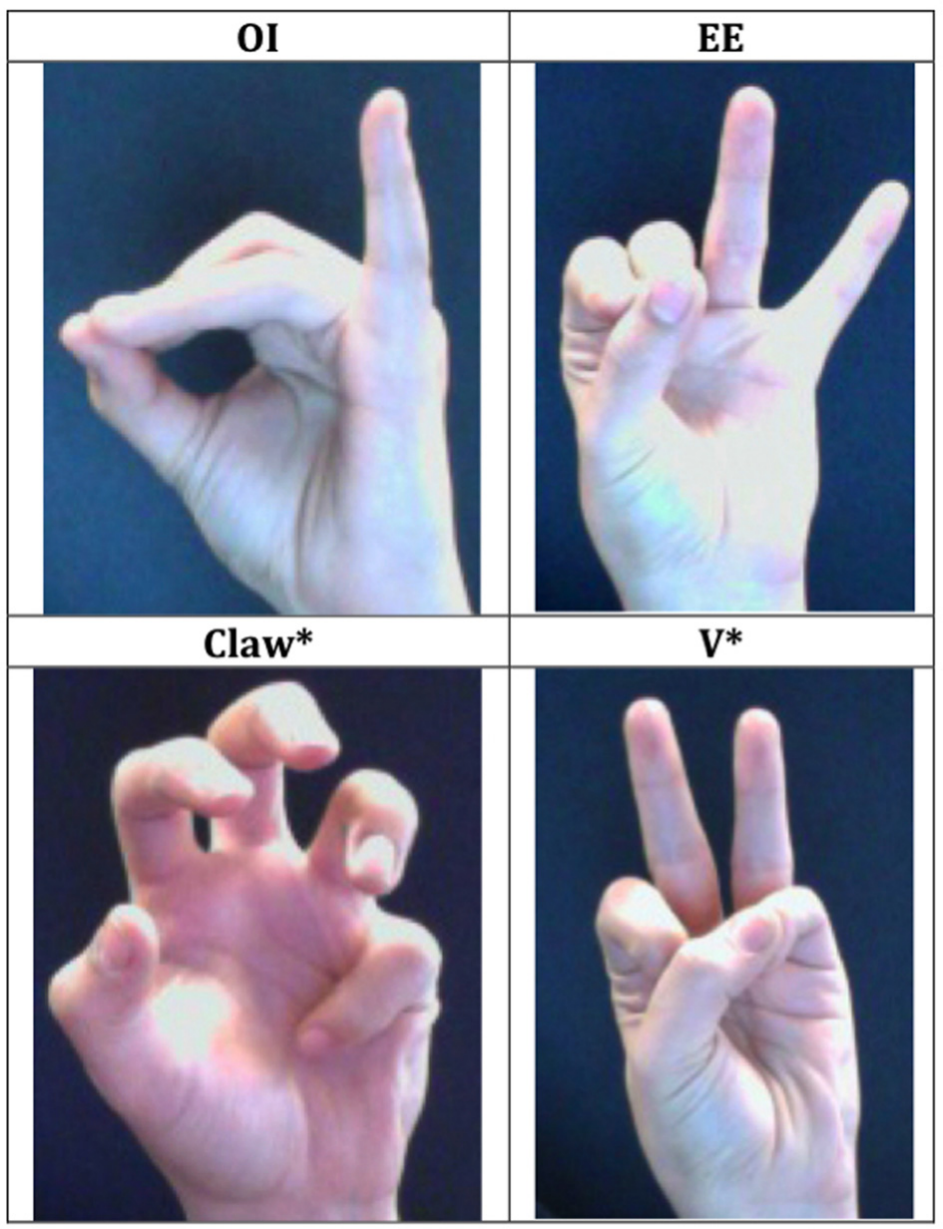
**An illustration of the four unattested handshapes used in Experiments 3–4**.

To test this possibility, Experiments 3–4 present participants with novel signs whose reduplicated syllable (X) includes a handshape that is unattested in ASL. Four such handshapes were selected: the OI, EE, V^*^ and the Claw^*^^8^ (see Figure 4). These four handshapes are all sign-like, and two of them—the OI and EE handshapes—are attested in Russian Sign Language and Japanese Sign Language. But despite their phonotactic legality, those handshapes are distinctly unattested in ASL, and as such, they are unlikely to readily assimilate to an ASL handshape. This characteristic of the stimuli is significant because past computational results have shown that algebraic rules are necessary to capture the reduplication of unfamiliar features, but they are not indispensable in generalizations to familiar features (Marcus, 1998; Berent et al., 2012b). If participants were to misperceive the unattested handshapes as ASL features, then generalizations to such features would not require reliance on algebraic rules. Our choice of nonnative features was designed to counter this concern.

**Figure 5:**
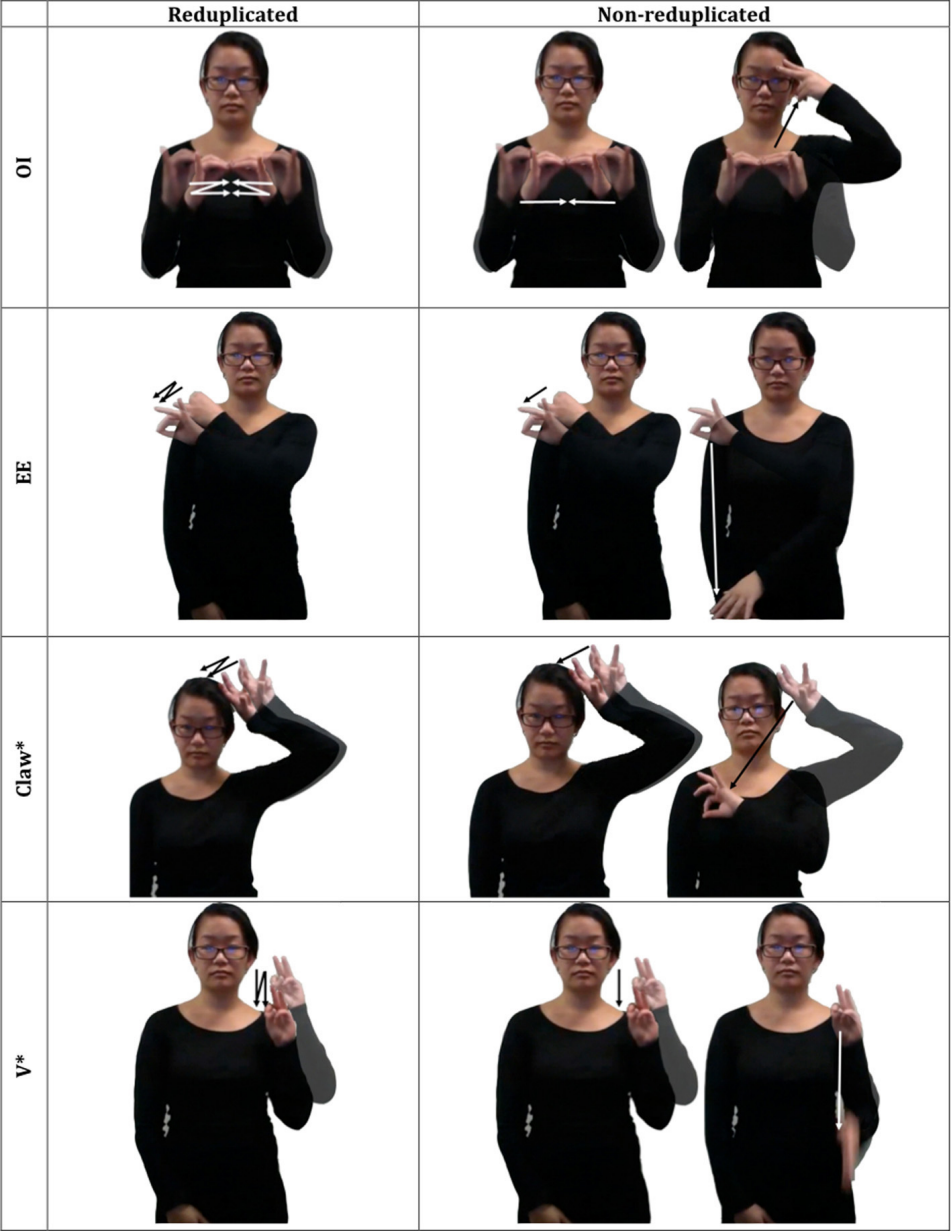
**An illustration of the novel signs with unattested handshapes used in Experiments 3, 4**.

Each such feature was incorporated in both a reduplicative novel sign (XX) and a nonreduplicative control (XY). In the XY controls, the initial syllable was identical to the reduplicated counterpart (XX), whereas the second syllable Y had a native handshape (see Figure 5). Note that the reduplicated signs were statistically less similar to ASL signs, as they included two unattested handshapes—more than in XY controls (with only a single unattested handshape). Accordingly, our experiments pit the contribution of the grammatical reduplication rule against the statistical structure of the ASL lexicon.

Experiment 3 first elicits off-line rating of novel XX and XY signs. To determine whether signers' preferences are informed by linguistic knowledge, we also obtained similar ratings from a group of English speaking nonsigners. Experiment 4 next examined whether signers extract reduplication on-line, in the lexical decision task.

### Experiment 3: off-line ratings

#### Methods

Participants were the same twelve Deaf adults and twelve English speakers who took part in Experiment 1 (rating novel ASL signs comprised of native features). Experiment 3 was administered after participants took part in Experiment 1.

***Materials.*** The materials consisted of short video clips, featuring sixteen novel pairs of ASL signs. Within each pair, one member was reduplicated (XX) whereas the other was nonreduplicated (XY), matched to its reduplicated counterpart for the initial syllable (X). In each such member, the syllable X comprised of a handshape that is unattested in ASL, whereas the Y syllable had a native ASL handshape. Four unattested handshapes were used: OI, EE, V^*^ and Claw^*^. The OI and EE handshapes are attested in Russian Sign Language and Japanese Sign Language; the remaining two handshapes were designed to appear as sign-like. Each such handshape was incorporated in four pairs.

All other features were matched to the novel signs employed in Experiments 1–2. Specifically, each unattested nonsign was created by replacing the handshape in syllable X of the attested nonsigns (used in Experiments 1–2) with one of the four nonnative handshapes mentioned above. Unattested nonsigns matched the attested nonsigns for location, movement, palm-orientation, and handshape in the Y syllable, and these items were thus phonotactically legal in ASL.

All video clips were recorded by a native ASL signer (the same individual featured in all experiments). Prior to the video recording, the signer practiced the signs, to ensure their fluent production. The video clips were subsequently edited, so that each clip began with the initiation of the signing movement and ended with the signer returning to a neutral position. All items were inspected for clarity by a fluent ASL signer (DB).

***Procedure.*** This was identical to Experiment 1.

#### Results

**Figure 6:**
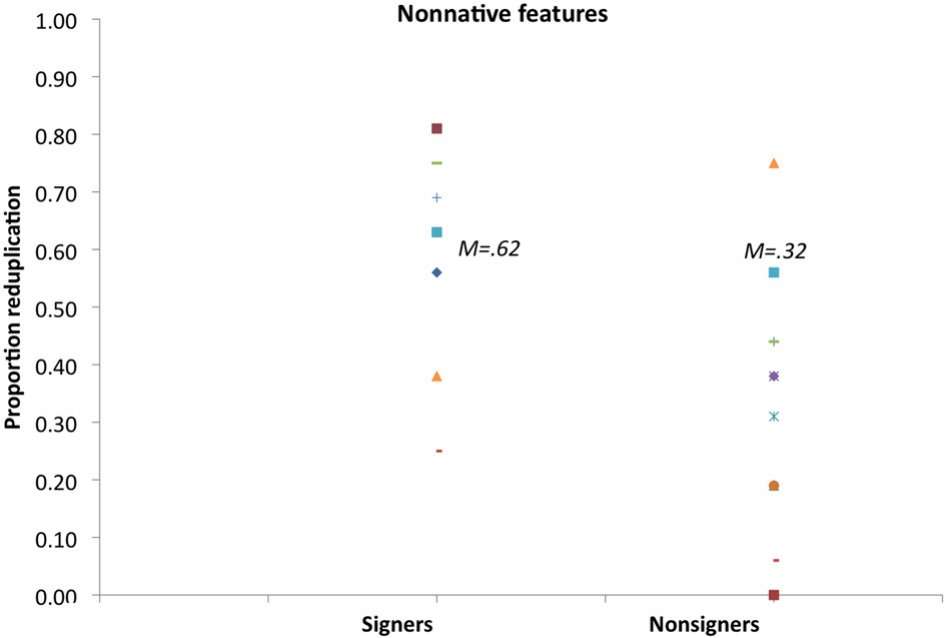
**Rating preference for reduplicated signs with unattested handshapes in Experiment 3**.

Figure 6 plots the proportion of trials in which participants favored the reduplicated sign over its nonreduplicated counterpart. An inspection of the means suggests that, on most trials, signers favored the reduplicated signs. T tests, assessing the reliability of this preference across participants' and items' means confirmed that preference for reduplicated signs was reliably different from chance level [*M* = 62%, *t*1_(11)_ = 2.48, *p* < 0.04; *t*2_(15)_ = 2.59, *p* < 0.03]. In contrast, nonsigners exhibited an opposite preference for nonreduplicated signs [*M* = 32%, *t*1_(11)_ = −2.86, *p* < 0.02; *t*2_(15)_ = −3.81, *p* < 0.002].

Signers' consistent preference for the reduplicated signs is remarkable given that these stimuli were statistically *less* similar to ASL signs than the nonreduplicative controls. Indeed, XX stimuli included two unattested ASL handshapes (one for each X syllable), whereas XY controls only had one such feature. The consistent preference for reduplication, despite conflicting statistical information, demonstrates that signers extracted the reduplicative structure. Their capacity to do so with unattested features could imply a productive algebraic rule.

### Experiment 4: lexical decision

In Experiment 4, we examine whether signers can extract the reduplication of unattested features in on-line language processing. To this end, we present the same set of novel signs from Experiment 3, mixed with ASL signs (used in Experiment 2) in a lexical decision task. Within each category, half of the stimuli were reduplicated, the others were nonreduplicated. In each trial, participants saw a single stimulus—either an ASL stimulus, or a novel sign with an unattested handshape.

Our experiment addresses two questions. First, we ask whether signers register the presence of unattested features in our materials. If they do, then novel signs with unattested features should be more readily recognized as such. Consequently, lexical decision in Experiment 4 should be faster and more accurate relative to Experiment 2—where the same ASL signs were paired with novel signs whose handshapes are attested in ASL.

Having demonstrated that participants registered the novel handshape faithfully, we can next move to examine our main question—whether signers represent its reduplication. If signers extract the reduplicative structure of novel handshapes, then novel XX signs should appear more sign-like (either because reduplication is less marked, or more frequent in ASL disyllables), hence, they should impair the identification of novel reduplicative signs relative to nonreduplicated controls.

#### Methods

Twelve Deaf adult, native ASL signers took part in the experiment. These individuals also took part in Experiment 2 prior to completing this experiment. Thus, the order of the four experiments was 2, 4, 1, 3 (i.e., rating and lexical decision for novel signs with attested features, followed by rating and lexical decision of novel signs with unattested features), and they were all administered in a single session. Materials, Instructions and Procedure were the same as in Experiment 2, except that the novel signs had unattested handshapes, as described in Experiment 3. The instructions to the experiment informed participants that they were about to see novel signs that do not occur in ASL, but contain elements that are borrowed from other sign languages.

#### Results

***Do signers register the presence of unattested handshapes?*** Before we can examine our main question of interest—whether signers are sensitive to the reduplication of unattested handshapes—we must first establish that signers did in fact register the presence of unattested features in our materials. If they did, then lexical decision should be easier to perform for nonsigns with unattested ASL features (in Experiment 4) compared to those with attested features (in Experiment 2).

To test this possibility, we compared the lexical decision responses in Experiment 4 (with unattested handshapes) to those in Experiment 2 (with attested handshapes) via 2 attestation (attested vs. unattested handshapes) x 2 lexicality (signs vs. novel signs) ANOVAs. As in Experiment 2, response time was inspected to eliminate outliers (correct responses slower than 3000 ms or faster than 250 ms, less than 1% of the total correct responses).

**Figure 7:**
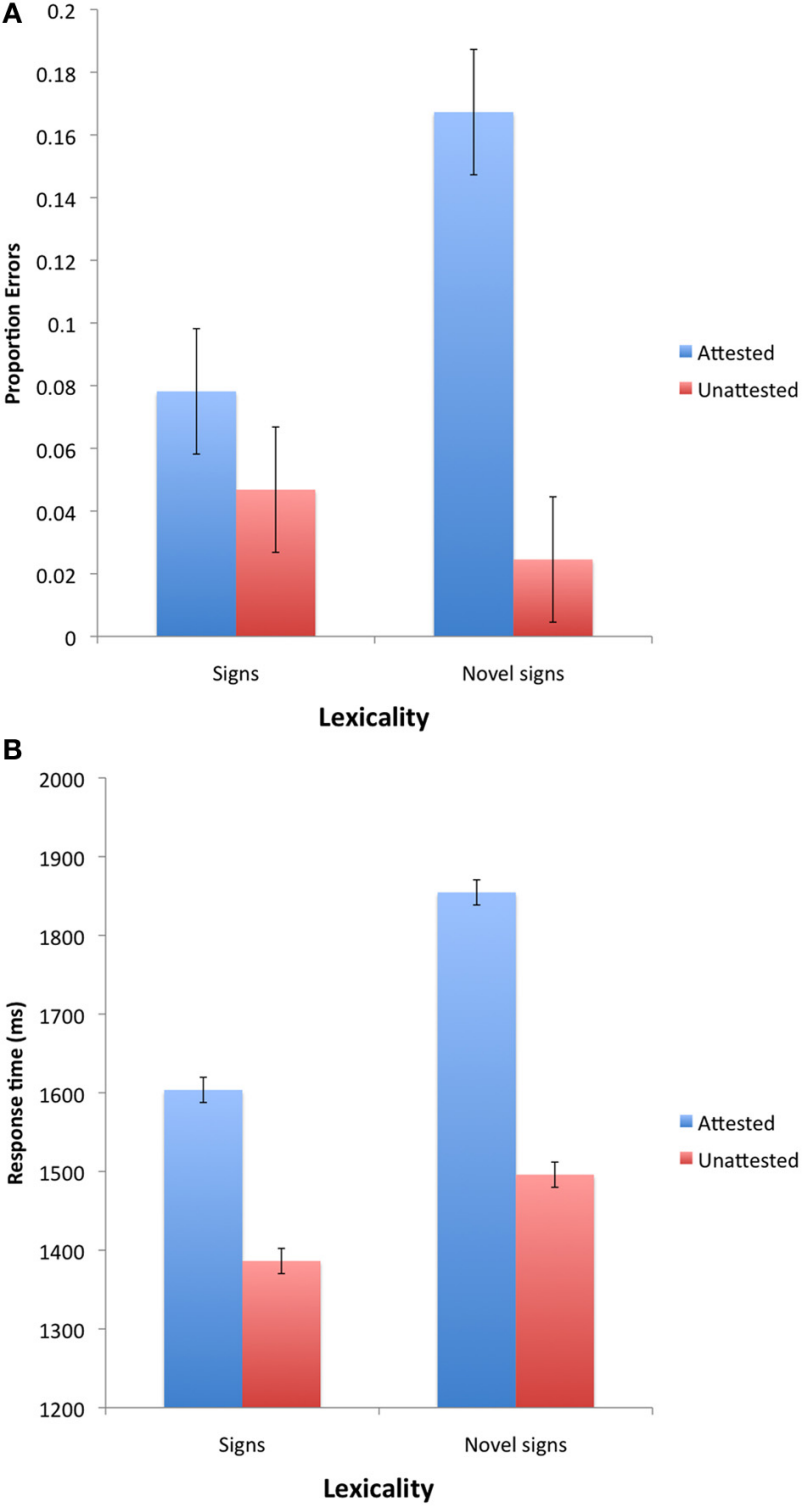
**The effect of handshape attestation on lexical decision across experiments**. Note: Error bars are 95% confidence intervals for the difference between the means.

An inspection of the means (see Figure 7) suggests that the unattested handshapes in Experiment 4 elicited faster and more accurate responses. While these savings were evident irrespective of lexicality, their magnitude was stronger for novel signs relative to ASL signs. Accordingly, the ANOVAs yielded reliable effects of attestation [In errors: *F*1_(1, 10)_ = 37.34, MSE = 0.003, *p* < 0.0002; *F*2_(1, 30)_ = 5.87, MSE = 0.076, *p* < 0.03; In response time: *F*1_(1, 10)_ = 42.17, MSE = 21,632, *p* < 0.00001; *F*2_(1, 30)_ = 31.67, MSE = 14,538, *p* < 0.00001] and lexicality [In errors: *F*1_(1, 10)_ = 4.45, MSE = 0.003, *p* < 0.07; *F*2_(1, 30)_ = 21.42, MSE = 0.033, *p* < 0.0001; In response time: *F*1_(1, 10)_ = 47.31, MSE = 7554, *p* < 0.00001; *F*2_(1, 30)_ = 343.92, MSE = 3841, *p* < 0.0001]. The interaction was significant in the analyses of response time [*F*1_(1, 10)_ = 34.80, MSE = 1576, *p* < 0.0002; *F*2_(1, 30)_ = 17.65, MSE = 3481, *p* < 0.0003], and marginally significant in errors [*F*1_(1, 10)_ = 15.1, MSE = 0.002, *p* < 0.004; *F*2_(1, 30)_ = 2.53, MSE = 0.033, *p* < 0.13].

Tukey HSD tests showed that responses to ASL signs were significantly faster in the presence of novel signs with unattested handshapes compared to ones with attested handshapes (*p* < 0.001, by participants and items). Likewise, novel signs with unattested handshapes elicited faster and more accurate responses relative to those with attested handshapes (*p* < 0.001, by participants and items).

Having established that participants did notice the presence of unattested handshapes, we can next ask whether they represented their reduplicative structure. To this end, we now turn to examine the effect of reduplication on responses to ASL signs and novel signs in Experiment 4.

***Are signers sensitive to the reduplication of unattested handshapes?***

**Figure 8:**
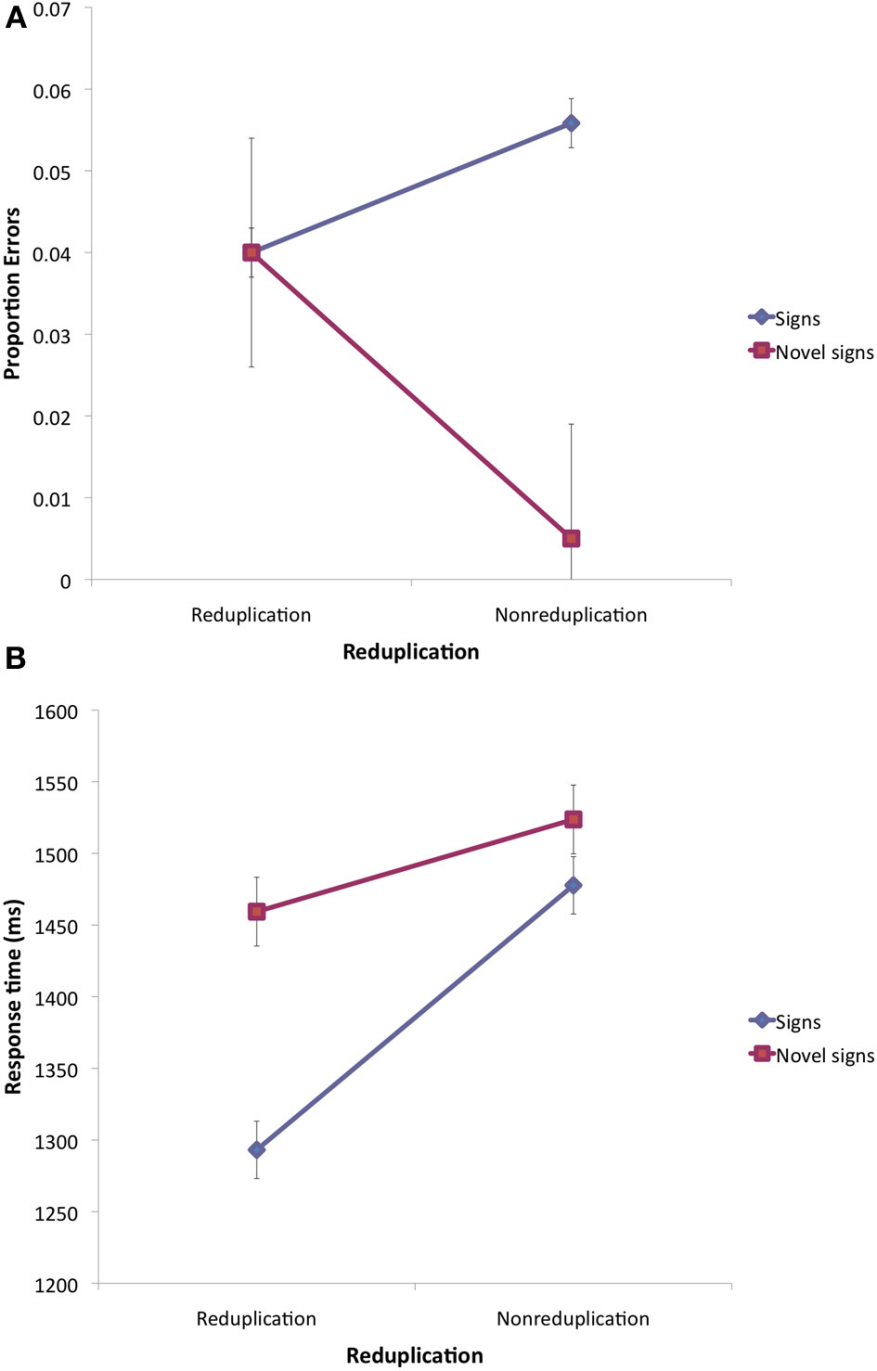
**Lexical decision results for ASL signs and novel signs with handshapes in Experiment 4**. Note: Error bars are 95% confidence intervals for the difference between the means.

*Errors.* An inspection of the means (see Figure 8) suggests that reduplication produced different effects for existing signs and novel signs. The 2 reduplication × 2 lexicality ANOVAs on the proportion of errors (arcsine transformed) only produced a marginally significant interaction [*F*1_(1, 11)_ = 4.89, MSE = 0.035, *p* < 0.05; *F*2_(1, 30)_ = 1.67, MSE = 0.058, *p* < 0.21]^9^.

A simple main effect analysis demonstrated that novel reduplicated signs elicited a significant increase in errors relative to nonreduplicated controls [*t*1_(11)_ = 2.73, *p* < 0.02; *t*2_(15)_ = 1.84, *p* < 0.05 one-tailed]. In contrast, for attested ASL signs, reduplication resulted in a nonsignificant decrease in errors (both *t* < 1).

*Response time.* An inspection of the means (see Figure 8) suggests that reduplication facilitated response time for both signs and nonsigns, although this effect appears more pronounced for attested ASL signs.

The 2 lexicality × 2 reduplication ANOVAs yielded reliable effects of lexicality [*F*1_(1, 11)_ = 12.82, MSE = 10,534, *p* < 0.005; *F*2_(1, 30)_ = 9.84, MSE = 17,765, *p* < 0.004], reduplication [*F*1_(1, 11)_ = 55.12, MSE = 3374, *p* < 0.0001; *F*2_(1, 30)_ = 23.06, MSE = 10,238, *p* < 0.0005] and their interaction [*F*1_(1, 11)_ = 16.98, MSE = 2557, *p* < 0.002; *F*2_(1, 30)_ = 6.05, MSE = 10,238, *p* < 0.02]. The simple main effect of reduplication was significant for both signs [*t*1_(11)_ = 9.19, *p* < 0.0001; *t*2_(15)_ = 4.65, *p* < 0.0004] and novel signs [*t*1_(11)_ = 2.66, *p* < 0.03; *t*2_(15)_ = 1.87, *p* < 0.05, one-tailed].

#### Discussion

The main finding of Experiment 4 is that signers are sensitive to the structure of novel signs with unattested ASL handshapes. First, participants had registered the presence of unattested handshapes, as their lexical decision responses in this experiment (i.e., in the presence of unattested handshapes) were reliably faster and more accurate relative to Experiment 2 (where all stimuli had handshapes that are native to ASL)^10^. Crucially, participants were sensitive to the reduplicative structure of these stimuli. Novel reduplicated signs produced a higher error rate compared to nonreduplicated controls. In contrast, reduplicated ASL signs elicited faster responses.

The selectivity of the effect of reduplication to the lexicality of the stimulus—whether it is an ASL sign or a novel sign—would appear to suggest that reduplicated signs are generally identified as more sign-like. Consequently, reduplication renders novel signs harder to classify as such. This conclusion, however, is countered by the finding that the response time saving associated with reduplication extended even for novel signs. Thus, for novel signs, reduplication elevated error rates, but sped up response time.

These conflicting effects of reduplication on response time and accuracy are amenable to two distinct explanations. One possibility is that reduplication incurs genuine savings in the processing of novel signs—perhaps because the redundancy facilitates their encoding by the visual system. Alternatively, the effect of reduplication could emanate from uncontrolled variations in the duration of these stimuli.

An inspection of the materials indeed showed that the duration of reduplicated stimuli were overall shorter than nonreduplicated stimuli for both ASL signs (*M* = 2024 ms, *M* = 2054 ms; for reduplicated and nonreduplicated signs, respectively) and novel signs (*M* = 2168 ms, *M* = 2201 ms; for reduplicated and nonreduplicated signs, respectively). While this difference may well reflect a systematic effect of reduplication on sign production, its presence confounds the effect of reduplication on perception.

To address this limitation, we assessed the effect of reduplication in a stepwise linear regression analysis, conducted separately for ASL signs and novel signs. Stimulus duration was forced into the model in the first step; reduplication was entered last. Results showed that, for existing ASL signs, the effect of reduplication remained highly significant, even after controlling for the effect of stimulus duration [*R*^2^_change_ = 0.318, *F*2_(1, 29)_ = 20.64, *p* < 0.0001] In contrast, once stimulus duration was controlled, the effect of reduplication on novel signs was no longer significant [*R*^2^_change_ = 0.049, *F*2_(1, 29)_ = 1.93, *p* < 0.18, n.s.]^11^.

Together, the results establish that reduplicated signs are identified as more sign-like. Existing ASL signs that exhibit reduplication are identified more rapidly than nonreduplicated controls. Crucially, reduplication exerts the opposite effect for novel signs. Once stimulus duration was controlled, reduplication did not affect response time, but it reliably elevated errors to novel reduplicated signs. These findings demonstrate that signers extracted reduplication of novel features that they have never encountered before. This conclusion is consistent with the possibility that ASL signers encode abstract algebraic rules.

## General discussion

Spoken languages include productive principles that allow speakers to extend their linguistic knowledge to novel instances (Chomsky, 1957). Across-the-board generalizations are significant because they are the hallmark of abstract algebraic rules (Fodor and Pylyshyn, 1988; Pinker and Prince, 1988; Marcus, 2001). Here, we asked whether such rules might also form part of the computational machinery of sign language. To this end, we examined whether signers can likewise extend their linguistic knowledge broadly.

As a case study, we examined signers' capacity to extend a reduplication rule—a rule that *inter alia* forms disyllabic nouns by reduplicating their monosyllabic verbal bases (X→XX). In four experiments, we asked whether signers extend reduplication to novel signs. Experiments 1–2 examined novel signs that reduplicate native ASL syllables; in Experiments 3–4, we probed for the reduplication of syllables whose handshape features are unattested in ASL. Given that reduplicated disyllables are favored in ASL (i.e., they are more frequent and possibly unmarked relative to nonreduplicated disyllables), we expected the reduplication rule to elicit a preference for novel reduplicated signs. This prediction was borne out in each of our four experiments. Experiments 1 and 3 showed that novel reduplicated signs are preferred to their nonreduplicative counterparts, and this preference obtained irrespective of whether the reduplicative feature is attested in ASL (in Experiment 1) or unattested (Experiment 3). Experiments 2 and 4 demonstrated that signers encode reduplication on-line, in lexical decision. In both experiments, novel signs with reduplicated features were more difficult to identify than their nonreduplicated counterparts, whereas reduplicated signs were identified more readily.

It is unlikely that the preference for reduplicated signs reflects a generic perceptual advantage. In fact, reduplicative signs were systematically dispreferred by nonsigners (in Experiments 1 and 3), and they were harder for signers to process (for novel signs, in Experiments 2 and 4).

The preference for reduplicated syllables is likewise inexplicable by their feature similarity (i.e., the fact that the XX syllables shared all their features, whereas XY syllables only shared some of those features). Our survey of nonreduplicative disyllables in the ASL lexicon reveals that partly similar signs—those in which the two syllables share location—are systematically *underrepresented* relative to dissimilar signs (i.e., those in which the location feature is not shared; for details, see footnote 12)^12^. Thus, acceptability (estimated by lexical frequency) is not a linear function of similarity (i.e., feature overlap): full identity is preferred, but partial similarity is systematically avoided—a result also found in spoken languages (e.g., Berent and Shimron, 2003; Berent et al., 2004). This conclusion counters the possibility that the preference for reduplicated signs (most critically, ones with unattested handshapes) is only due to the partial similarity among some of their native features. Further evidence against this possibility is presented by responses to the nonreduplicative disyllables in our experiments. Had the preference for XX signs been solely due to the (partial) overlap among their native features, then feature overlap should have predicted the acceptability of nonreduplicative XY signs—novel XY with greater feature overlap should have appeared more sign-like, hence, harder to identify as novel signs. However, our results yield no correlation between the acceptability of novel XY signs (across Experiments 2 and 4) and their feature similarity [*r*_(30)_ = 0.08, for both accuracy and response time]. Given that partial similarity appears to be dispreferred (as judged by its underrepresentation in the lexicon), the preference for XX signs must be specifically due to the full identity of their syllables, including their unattested handshape.

Another similarity-based explanation attributes the preference for XX signs to the statistical properties of the ASL lexicon. But this explanation is also inconsistent with the available evidence. Recall that in Experiments 3–4, XX signs were favored to XY controls despite having two unattested handshapes (compared to only one unattested handshape in the XY controls). Thus, the preference for reduplicated signs is irreducible to their feature similarity to ASL signs. It is also unlikely that novel XX signs had larger neighborhoods than XY signs. By definition, XX signs with two unattested handshapes have no neighbors at all, as a neighbor differs from the target on a single parameter (Baus et al., 2008; Carreiras et al., 2008). Likewise, the neighborhoods of our attested signs were extremely sparse, as only two of our items had a neighbor (one reduplicated, with a single neighbor, and one nonreduplicated, with two neighbors). These observations offer no support for the lexical similarity account. Given that the preference for XX syllables is inexplicable by either the feature similarity among their two syllables or their statistical similarity to the ASL lexicon, the most likely explanation for our results is that the preference for XX signs reflects their reduplication.

Our findings show for the first time that signers' knowledge of their native language supports systematic generalizations that extend across the board—even to features that they have never encountered before. Algebraic rules provide a natural computational explanation for these findings. Because such rules operate on variables that stand for entire equivalence classes (e.g., any syllables), algebraic rules apply broadly, irrespective of the familiarity with novel items and their similarity to familiar stimuli.

Not only are these results consistent with the encoding of algebraic rules, they are also inconsistent with a nonalgebraic alternative. Past computational simulations, attempting to capture reduplication rules using nonalgebraic mechanisms (i.e., mechanisms that lack the capacity to operate over variables)—either connectionist networks (Marcus, 1998, 2001), or a state of the art inductive learner (Berent et al., 2012b)—have failed to adequately capture human generalizations. As in the present experiment, these simulations examined generalization of an identity function to test items including a single unattested feature. Results showed that, absent operations on variables, these models failed to generalize to such items. While the capacity of such models to account for the present data remains to be seen, the close parallels with previous test cases from spoken language suggest that their success for reduplicated signs is unlikely. Accordingly, signers' capacity to extend reduplication across the board suggests that their linguistic knowledge of reduplication relies on algebraic rules.

The conclusion that the ASL grammar encodes algebraic rules does not speak to the precise nature of rules available to participants. While our materials were modeled after the morphological rule that obtains nouns from verb reduplication, these results cannot determine whether signers effectively represented the novel reduplicative signs as nouns. We also note that our evidence for rules does not negate the possibility that some aspects of linguistic knowledge are associative, or even iconic (Ormel et al., 2009; Thompson et al., 2009, 2010, 2012). While these alternative representations and computational mechanisms might be ultimately necessary to offer a full account of the language system, our present results suggest that they are not sufficient. At its core, signers' phonological knowledge includes productive algebraic rules, akin to the ones previously documented in spoken language phonology. These results suggest that the computational architecture of the phonological mind is at least partly amodal (Berent, 2013a,b).

## Author notes

We wish to thank Krista Lavrentios and Livymer Caceres for their assistance in running the participants in this study.

## Conflict of interest statement

The authors declare that the research was conducted in the absence of any commercial or financial relationships that could be construed as a potential conflict of interest.

## Footnotes

^1^Algebraic rules, as discussed here, are distinct from the technical definition of linguistic rules (mappings from inputs to outputs)—a notion that contrasts with constraints (operations over outputs). Indeed, linguistic rules and constraints both apply to structured representations by virtue of their constituent structure, and as such, they both invoke the same notion of algebraic rules examined here.

^2^The potential of a rule to apply across the board does not mean that the operation of the rule is never circumvented. The English plural rule, for instance, is blocked whenever irregular counterexamples are retrieved from memory. Such limitations, however, are imposed by factors external to the rule (e.g., conflicting rules, lexical stipulations), rather than from limitations on the inherent capacity of the rule to generalize, and as such, they are irrelevant to evaluating the scope of potential generalizations.

^3^The algebraic account is further challenged by the iconicity of signs (Ormel et al., 2009; Eccarius and Brentari, 2010; Thompson et al., 2010; Brentari, 2011), which has been shown to affect their on-line processing by adults (Thompson et al., 2009, 2010), children (Ormel et al., 2009) and infants (Thompson et al., 2012; but see Emmorey et al., 2004; Bosworth and Emmorey, 2010). Iconicity implies that the representation of signs is continuous and analog, not discrete and digital, as required by the algebraic proposal. However, the effects of iconicity are not specific to sign language (for a recent review of spoken language, see Schmidtke et al., 2014). Moreover, the encoding of phonetic and embodied aspects of signed and spoken words does not preclude the existence of a second format of representation that is algebraic, abstract and fully productive (Brentari, 2007; Mahon and Caramazza, 2008; Eccarius and Brentari, 2010).

^4^It has been argued that the targeted syllables are “light” (i.e., syllables with a single movement component, Brentari, 1998).

^5^Marked structures are (a) disfavored as the output of grammatical processes (de Lacy, 2006); and (b) underrepresented in the language (Prince and Smolensky, 1993/2004). Reduplicated signs meet both requirements for (un)markedness. While many grammatical processes produce reduplicated signs (Klima and Bellugi, 1979; Wilbur, 2009), nonreduplicated disyllables are avoided, resulting in their reduction to monosyllables (Sandler and Lillo-Martin, 2006). In addition, nonreduplicated disyllables are systematically underrepresented in the ASL lexicon. Our inspection of an on-line ASL dictionary (ASLpro.com) identified a total count of 1830 disyllables. Of those, the grand majority (69.7%) are fully reduplicated, 20% are partially reduplicated whereas only 10.27% are nonreduplicated. Given those observations, XX signs are likely less marked than XY ones.

^6^Some of these ASL signs also have a one movement variant, but these monosyllabic variants were not the ones used in our experiment—all experimental items were invariably disyllables with two full movements.

^7^To ensure that the error results are not due to artifacts associated with binary data, we also submitted the error data to a mixed effects logistic analysis, with lexicality and reduplication as fixed effects (sum-coded) and participants and items as random effects. These analyses yielded a reliable lexicality × reduplication interaction (β = −0.6061, *SE* = 0.122, *Z* = −4.95, *p* < 0.001).

^8^We use the asterisk to distinguish the novel V and Claw handshapes from the V and Claw handshapes in ASL, rather than the typical indication of ill-formedness.

^9^The interaction was likewise reliable in the logit analysis (β = −0.627, *SE* = 0.319, *Z* = −1.97, *p* < 0.05).

^10^The ease of discrimination in Experiment 4 is unlikely to reflect a simple practice effect (due to its administration after Experiment 2) as a median split analysis of response accuracy in Experiment 2 and 4 according to trial order (first vs. second half) found no reliable effects of block order (*t* < 1).

^11^Another alternative explanation attributes the effect of reduplication to uncontrolled variation in movement repetitions. Since some of our nonreduplicated (XY) controls did not share the same movement type in their X and Y syllables, the co-occurrence of two identical movement types could have rendered novel reduplicated signs more sign-like. Most (11/16) item pairs, however, did share the same movement type. Moreover, a comparison of item pairs that shared the same movement type with those that did not (via a 2 movement × 2 reduplication ANOVA) found no effect of movement repetition on response accuracy (all *F* < 1). Accordingly, the effect of reduplication is unlikely due to the type of movement alone.

^12^To determine whether partial feature similarity is preferred, we extracted from an on-line ASL dictionary (ASLpro.com) all disyllabic signs whose two syllables are nonreduplicative—a total of 366 signs. To isolate the effect of feature overlap along a single parameter—location—we further limited the search to nonreduplicative signs whose syllables do not share a handshape—a total of 188 signs. We next coded each such sign for the location of its two syllables along ten different location categories (mouth, neutral, head, contact with non-dominant hand, chest, arm, ear, face, chin, torso), and indicated whether or not the two syllables share the same location. Of the 188 signs surveyed, only 33 signs (i.e., 0.175) shared location—a proportion that is unexpected by the chance level of 0.5 (*p* < 0.0001 by a binomial test). This finding demonstrates that, in the absence of full identity, partial feature similarity is actively avoided in the ASL lexicon. This finding is inconsistent with the possibility that the preference for reduplicated signs in our experiments is due to the partial feature overlap among the two syllables.
