# Supplementary material for: Phonological reduplication in sign language: Rules rule
Source: Front Psychol. 2014 Jun 10;5:560. doi: 10.3389/fpsyg.2014.00560 (PMC4050968; doi:10.3389/fpsyg.2014.00560)
Supplement: Supplementary file 1 [file DataSheet1.DOCX]

**Appendix A**. The matched syllables used in the novel signs with native features.

Note: shown are the matched X and Y syllable in each item pair. Pair members were generated by either reduplicating the X syllable (in reduplicated signs) or concatenating the X and Y syllables (in nonreduplicated signs).

**Appendix B** The ASL signs used in Experiments 2 and 4^[[1]](#footnote-1)^. The English translation of nonreduplicated sign is provided; for reduplicated signs, the translation is identical to the gloss provided below.

| **Item** | **Reduplicated** | **Non-reduplicated** | **English Translation** |
| --- | --- | --- | --- |
| 1 | CHAIR | BLACK-NAME | “bad reputation” |
| 2 | DOOR | BLUE-SPOT | “bruise” |
| 3 | WINDOW | DEAF-CLOSE | variant of “deaf” |
| 4 | BOOK | TEACHER | “teacher” |
| 5 | JAR-LID | MIND-DROP | “shock” |
| 6 | KEY | RED-BLOOD | variant of “blood” |
| 7 | HEARDING AID | DECIDE | “decide” |
| 8 | CAR | FACE-STRONG | “resemblance” |
| 9 | SCISSORS | OVER-SLEEP | “over sleep” |
| 10 | ZIPPER | HEART-ATTACK | “heart attack” |
| 11 | EARRING | SLEEP-CLOTHES | “pajamas” |
| 12 | HAT | BELIEVE | “believe” |
| 13 | PLUG | EAT-NIGHT | “dinner” |
| 14 | GAS | EAT-MORNING | “breakfast” |
| 15 | TELEPHONE | SICK-SPREAD | “infect (other people)” |
| 16 | AIRPLANE | STUDENT | “student” |

**Appendix C**. The matched syllables used in the novel signs with handshapes that are unattested in ASL.

Note: shown are the matched X and Y syllable in each item pair. Pair members were generated by either reduplicating the X syllable (in reduplicated signs) or concatenating the X and Y syllables (in nonreduplicated signs).

1. Some of the non-reduplicated ASL signs have both a monosyllabic and disyllabic version in ASL. Here, we invariably used disyllabic signs. [↑](#footnote-ref-1)
